# Production of chitin and bioactive materials from Black tiger shrimp (*Penaeus monodon*) shell waste by the treatment of bacterial protease cocktail

**DOI:** 10.1007/s13205-014-0245-6

**Published:** 2014-08-28

**Authors:** Tanamy Paul, Suman K. Halder, Arpan Das, Kuntal Ghosh, Arpita Mandal, Pijush Payra, Prasenjit Barman, Pradeep K. Das Mohapatra, Bikas Ranjan Pati, Keshab C. Mondal

**Affiliations:** 1 Department of Microbiology, Vidyasagar University, Midnapore, 721102 West Bengal India; 2 Department of Aquaculture Management and Technology, Vidyasagar University, Midnapore, 721102 West Bengal India

**Keywords:** Black tiger shrimp shell wastes, *Paenibacillus woosongensis*, Cocktail protease, Chitin, Amino acid

## Abstract

The main objective of this study was to obtain chitin in pure form from a new crustacean waste material for industrial applications. Black tiger shrimp shell wastes are a rich source of protein and valuable bioactive carbohydrate polymers such as chitin. After removal of carotenoid, Black tiger shrimp shell wastes (BTSHWs) were treated with chemicals and protease enzyme to extract chitin. Box–Behnken response surface methodology was applied to optimize the deproteinization process to obtain chitin. At optimal pH (8.82), temperature (50.05 °C), agitation speed (100.98 rpm), enzyme substrate ratio of 1:8 (wv^−1^) and 72 h of incubation with *Paenibacillus woosongensis* TKB2 crude protease cocktail, 80 % deproteinization was found along with 77.28 % recovery of chitin. The valuable oligopeptides were determined by MALDI-TOF analysis and analysis of adequate amount of free amino acids in protein hydrolysate from BTSHW, indicating a high nutritional value used for food, feed or as a nitrogen source in growth medium for microorganisms. The chitin obtained was compared with the commercial chitin using scanning electron microscopy, Fourier transform infrared spectrometer, X-ray diffraction and ^13^C CP/MAS-NMR. Chitin obtained from crude protease treatment showed comparable physicochemical and structural properties to those of the commercial chitin. The carotenoid obtained after treatment can be used for medicinal purpose.

**Electronic supplementary material:**

The online version of this article (doi:10.1007/s13205-014-0245-6) contains supplementary material, which is available to authorized users.

## Introduction

Chitin, one of the most abundant renewable biopolymers on earth, is a linear chain molecule composed of several hundred units of (1-4)-2-acetamido-2-deoxy-β-d-glucan. Among the natural chitinous resources, fishery wastes especially shrimp and crab shells have the highest content followed by the exoskeleton of arthropods and the fungal cell walls. Except that some are processed to make cheap feeds on shrimp and crab shell powder, most wastes are discarded at will (Wang et al. 1997). In consideration of the amount of chitin produced annually all over the world, it is the most copious renewable natural resource after cellulosic substrate (Rinaudo 2006). Chitin has many unique functional properties including biocompatibility, biodegradability and non-toxicity for which it has been widely applied in the sectors of food, agriculture, medicine, and materials (Muzzarelli et al. 2012; Franco and Peter 2011; Ling et al. 2011). Now a days, chemical (Abdou et al. 2008; Chandumpai et al. 2004), enzymatic (Valdez-Pena et al. 2010; Nakagawa et al. 2011) and microbiological methods (Ghorbel-Bellaaj et al. 2011; Jung et al. 2007) have been applied to prepare chitin from shrimp shell powders (SSPs). However, the use of these chemicals can seriously pollute the ecological environment, produce abundant waste, and damage human health. Moreover, the addition of acid and alkali can hydrolyze the polymer, resulting in inconsistent physiological properties of the final product. Along with increased demands on environment-friendly society and rapid development of fermentation technology, more eco-friendly processes using enzymatic and microbiological methods for producing chitin have attracted great interests. The enzymatic method includes the use of trypsase, papain, and pepsase (Valdez-Pena et al. 2010). After enzymatic treatment, the experimental liquor is found to contain high amounts of protein and amino acids, indicating a high nutritional value which can be used for food, feed or as a nitrogen source in growth media for microorganisms (Gildberg and Stenberg 2011). In fact, the potentiality of chitin as a storehouse of proteins and amino acids has prompted the present researchers to undertake the study.

As mentioned above, the objectives of this work were to investigate the influence of several operating parameters such as, enzyme/substrate ratio, temperature, pH, agitation speed and incubation time on their efficacy of deproteinization (DP) and demineralization (DM) degree of shrimp shells by non-commercial *P. woosongensis* TKB2 crude cocktail enzyme. RSM has employed to optimize these process parameters better than classical one-variable-at-a-time experimentation. Physicochemical and structural properties of extracting chitin were evaluated by scanning electron microscopy (SEM), Fourier transform infrared spectrometer (FT-IR), X-ray diffraction (XRD) and ^13^C CP/MAS-NMR analysis. In this work, we have given an HPLC analysis of free amino acids present in lyophilized protein fraction from shrimp shell waste hydrolysate obtained by enzymatic process. To the best of our knowledge, no studies have reported the preparation of chitin from BTSHW using crude protease. That is why, emphasis has been laid on enzymatic procedure on lieu of treatment with acid or alkali which is at all not eco-friendly practices when it is discarded in the surroundings.

## Experimental

### Raw materials and microorganism

The black tiger shrimp (*Penaeus monodon*) shells were obtained in fresh condition from the local market of Midnapore, West Bengal, India. Prior to use, the black tiger shrimp shells were washed thoroughly with distilled water. The shells were ground in a mixture grinder (Bajaj-Bravo, India) and the resulting small pieces of shells were used as raw materials (BTSHW). Casein, Bovine serum albumin and commercial chitin were supplied by HiMedia Co., India. All the other chemicals were of analytical and HPLC grade.

*Paenibacillus woosongensis* TKB2, a keratinase producing strain (Paul et al. 2013), was used for this study.

### Culture medium and enzyme production

*Paenibacillus woosongensis* TKB2 was grown in 50 ml of liquid medium in a 250 ml Erlenmeyer flask containing 0.75 % (wv^−1^) chicken feather, 0.05 % K_2_HPO_4_, 0.025 % MgSO_4_, 5 % NaCl, 0.020 % CaCO_3_, and 0.015 % FeSO_4_. The incubation temperature and pH were 30 °C and 8.5, respectively. After the culture was incubated for 48 h, the culture broth was centrifuged at 4 °C for 20 min at 10,000 rpm followed by the membrane filter (0.45 µm, Millipore, USA). The filtrate containing the crude enzyme was used in tests for Black tiger shrimp shell waste deproteinization.

### Protein estimation and zymogram analysis

Protein content in fermented liquor of BTSHW and solid BTSHW after fermentation from different periods of time was measured according to Lowry et al. (1951). SDS–PAGE 10 %, polyacrylamide containing 1 % casein (HiMedia, India) was performed for zymogram analysis of crude enzyme extract. The gel was stained with Coomassie brilliant blue R-250 (Mark, India).

### Separation of carotenoid from BTSHW

Extraction and separation of carotenoid from shrimp shell waste were investigated by application of the methods of Sindhu and Sherief (2011) using hexane: isopropanol 3:2 (vv^−1^) as organic solvent.

A known weight of homogenized wet and dry shrimp shell waste (1 g) was extracted with 10 ml of solvent to assess carotenoid recovery. The carotenoid extract was filtered using Whatman No. 2 filter paper. Recovered shrimp waste was repeatedly extracted with fresh solvent until the filtrate was colorless, to a maximum of three times. The pooled extracts were phase separated with equal volume of 1 % (w/v) NaCl solution. The appeals were collected and dehydrated with anhydrous sodium sulfate, and then evaporated to dryness under vacuum and the residue was dissolved in a 5 ml of hexane. The carotenoid was quantified in the Black tiger shrimp shell extract of the present study at 470 nm in hexane. The yield of the carotenoids was calculated as astaxanthin (Simpson and Haard 1985) using the following equation:

1 $${\text{Carotenoid yield }}\left( {\upmu {\text{g astaxanthin}}/{\text{g sample}}} \right) = \;\frac{{A_{{468 \, \text{nm} }} \times V_{\text{extract}} \times \text{Dilution} \, \text{factor} }}{{0.2 \, \times \, W_{\text{sample}} }}$$

where, *A* is absorbance, *V* is volume of extract, 0.2 is the *A*_468_ of 1 µg ml^−1^ of standard astaxanthin and *W* is weight of sample in grams.

After separation of carotenoid from BTSHW, solid residual BTSHW was used for deproteinization experiments.

### Deproteinization (DP) of Black tiger shrimp shell Waste (BTSHW)

Deproteinization was expressed as percentage and computed by the following equation as described by Rao et al. (2000).

2 $$\% \ \text{Deproteinization} \, = \, \left[ {\left( {P_{\text{O} } \times O} \right) - \left( {P_{\text{R} } \times R} \right)} \right] \times 100/P_{\text{O} } \times O$$

where, *P*_O_ and *P*_R_ are protein concentrations (%) before and after hydrolysis; while, *O* and *R* represent the mass (grams) of initial sample and hydrolyzed residue on dry weight basis, respectively.

### Acid treatment of BTSHW

BTSHW was mixed with 2 N HCl solution at a ratio of 1:8 (wv^−1^). The mixture was stored at 50 °C temperature for 4 days (Wang et al. 1997), and then filtered. After filtration, the solid residues were washed with de-ionized water until a neutral pH was obtained.

### Alkali treatment of BTSHW

BTSHW was mixed with 2 N NaOH solution at a 1:8 (wv^−1^) ratio. The mixture was allowed to react at 50 °C for 4 days (Wang et al. 1997) and then it was centrifuged. After centrifugation, the supernatant was used for analysis of protein concentration.

### Crude enzymatic treatment of BTSHW

The culture supernatant containing crude proteases (1.57 mg ml^−1^ of 71.4 U ml^−1^) obtained from the liquid-phase fermentation described above was tested for deproteinization. BTSHW was mixed with an enzyme solution at a 1: 8 (w/v) ratio. After the substrates were incubated with the protease solution at 50 °C for 4 days, the solid residues were isolated and washed, followed by deproteinization analysis of fermented liquor. The protein content was analyzed for the calculation of the protein removal rate.

### Commercial enzyme treatment of BTSHW

For comparison, one other commercially available protease (Sigma-Aldrich, USA) was also studied for shrimp shell waste deproteinization capabilities. The same experimental procedure was employed for this protease with same incubation temperature for 4 days.

### Demineralization (DM)

Demineralization was carried out in a dilute 1.25 M HCl solution. Solid fractions obtained after hydrolysis with different processes were treated with 1.25 M HCl in 1:10 (wv^−1^) ratio for 6 h at room temperature under constant stirring. The chitin product was filtered through four layers of gauze with the aid of a vacuum pump and washed to neutrality with de-ionized water and then freeze-dried. The residual minerals were estimated by HPLC (Agilant-1200, USA).

### Optimization of deproteinization process

To investigate the most contributing factors and the suitable level of each contributing factor in the deproteinization using a crude enzyme, response surface experimental design (RSM) was employed. The Box–Behnken design with three factors and three levels (+1, 0, −1), including three replicates at the centre point, was used for fitting a second-order response surface. A polynomial quadratic equation was applied to evaluate the effect of each independent variable to the response:

3 $$\text{Y} = \,\upbeta_{0} + \sum \,\upbeta_{i} \text{x}_{i} + \, \sum\upbeta_{ij} \text{x}_{i} \text{x}_{j} + \, \sum\upbeta_{ii} \text{x}^{2}_{i}$$

where, Y is the predicted response, x_*i*_ and x_*j*_ are the independent input variables in coded values that influence the response variable Y, β_o_ the offset term, β_*i*_ represents the linier effect of x_*i*_, β_*ij*_ represents the interaction effect between x_*i*_ and x_*j*_, β_*ii*_ represents the quadratic effect of x_*i*_. For statistical calculations, the variables X_*i*_ were coded as x_*i*_ according to the following equation:

4 $$\text{x}_{i} = \, \left( {\text{X}_{i} - \text{X}_{0} } \right)/ \,\updelta\text{X}$$

where, x_*i*_ is the dimensionless coded value of the variable X_*i*_, X_0_ is the value of the X_*i*_ at the centre point and δX is the step change. Each experimental design was carried out in triplicates.

### Analysis of chemical properties of sample

The moisture and ash content were estimated by the standard AOAC (2005) methods. Total nitrogen present in shrimp shell protein hydrolysate was determined using the Kjeldahl method. Total protein was estimated by multiplying total nitrogen content in chitin by the factor of 6.25. The BTSHW powder was analyzed for the selected minerals (Ca, Na, K and Mg) by high-performance liquid chromatography (Agilant-1200, USA).

### Scanning electron microscopic analysis

SEM (JEOL JSM5800, Japan) was used to clarify the superficial characteristics of the samples at 2,000× magnification. The samples were fixed on a sample holder and coated with a thin gold layer of 3 mm by a sputter coater (POLARON-SC7620) for conductivity. Shrimp shell wastes from different treatments were used for surface analysis through SEM and comparative study with commercial chitin.

### Fourier transform infrared spectroscopy (FT-IR) analysis

Fourier transform infrared spectroscopy (PerkinElmer-L160000A, USA) was used to record infrared spectra of samples between 4,000 and 500 cm^−1^. The degree of acetylation (DA) was determined using the following equation (de Vasconcelos et al. 2011).

5 $$\text{DA} \, \left( \% \right) \, = 115 \, \times \, \left( {A_{1,655} /A_{3,450} } \right)$$

where, *A*_1,655_ and *A*_3,450_ were the absorbances of samples at wave numbers of 1,655 and 3,450 cm^−1^, respectively. All samples were analyzed three times and DA values were reported as mean ± standard deviation.

### X-ray diffraction

The wide-angle X-ray diffraction (WAXD) analysis was applied to detect the crystallinity of commercial chitin in comparison with chitin prepared by crude protease treatment and their patterns were recorded using a Rigaku Americas, 9009 (Rigaku Co., USA). 2θ was scanned from 5 to 50° at a coating time of 2 s with an angle step width of 0.05°. The crystallinity index (Cr_Ipeak_) was calculated as (de Vasconcelos et al. 2011).

6 $$\text{Cr}_{\text{Ipeak}} = \, \left( {I_{110} {-}I_{\text{am}} } \right)/I_{110},$$

where *I*_110_ was the maximum intensity (arbitrary units) of the (110) lattice diffraction pattern at 2θ = 20° and *I*_am_ was the intensity of amorphous diffraction in the same units at 2θ = 16°.

## ^13^C CP/MAS-nuclear magnetic resonance (NMR) study

Chitin structural analysis was carried out by ^13^C NMR with CP/MAS technique (cross-polarization, magic-angle spinning) by BRUKER-ASX300 instrument. NMR spectra were recorded at a ^13^C frequency of 75.5 MHz (field of 7.04 T). CP/MAS sequence was used with the following conditions: the ^13^C spin lattice relaxation time was 5 s; powdered samples were positioned in an alumina rotor used for the double air-bearing-type MAS system and spun as fast as 8 kHz. Contact time was 8 ms.

### Oligopeptides and Amino acid quantification of Black tiger shrimp shell hydrolysate

After incubation for 4 days, the shrimp shell waste was centrifuged to collect the chitin fraction (sediment) and the protein-rich liquor (liquor). The protein liquor was filtered through 5 kDa Biomax polyethersulfone (PES) cutoff membrane using Amicon Stirred Ultrafiltration Cells (MA, USA), lyophilized. The samples were desalted by Zip Tip pipette chips containing C18-reverse-phased media (Millipore, Billerica, MA, USA) according to the supplier’s protocols before applying to a matrix-assisted laser desorption ionization-time of flight (MALDI-TOF) spectrometer. α-Cyano-4-hydroxycinnamic acid (10 mg ml^−1^) was prepared in 50 % acetonitrile/0.05 % trifluoroacetic acid as the matrix solution. Oligopeptides estimation was carried out according to Kumar et al. (2008) using Bovin serum albumin as a standard.

**Fig. 1:**
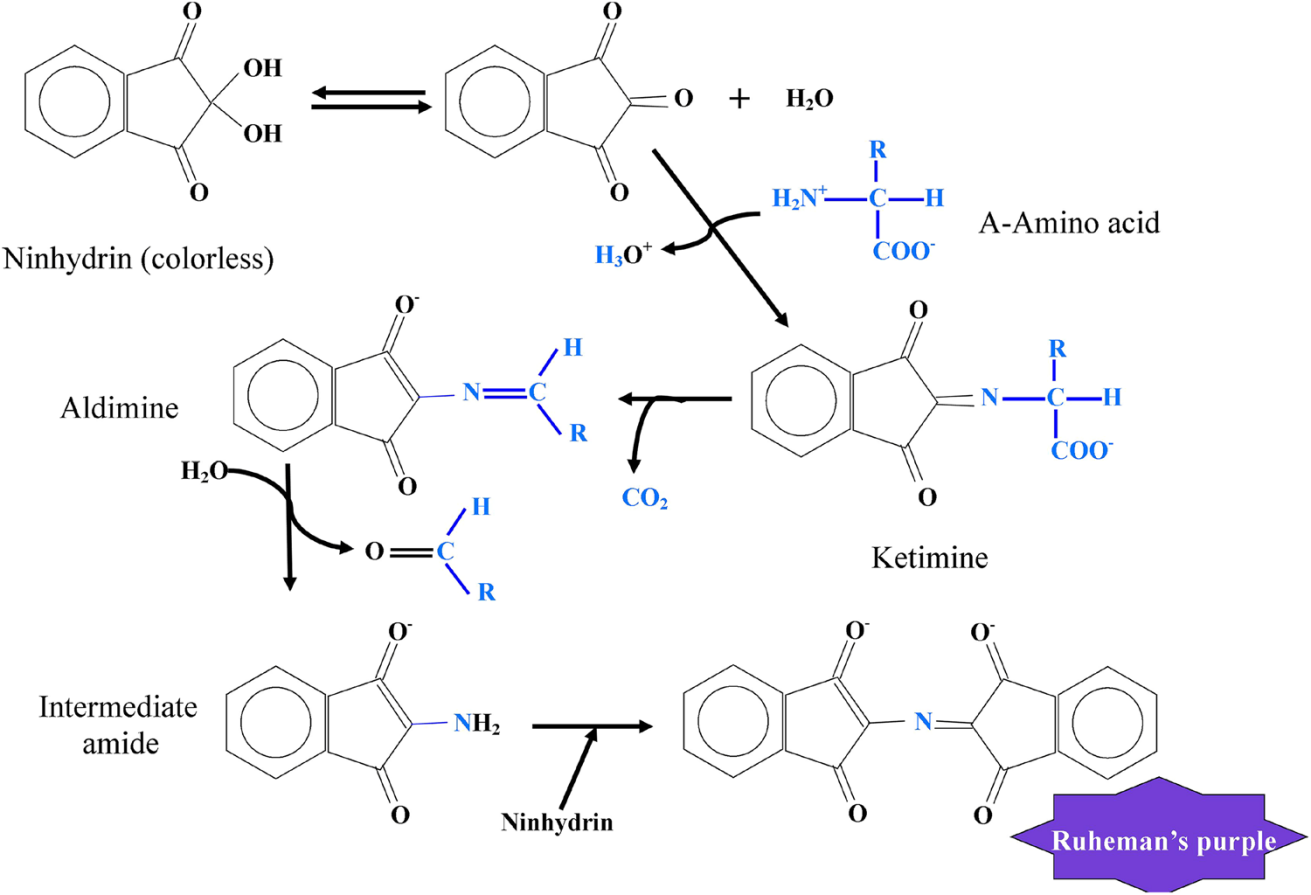
Schematic representation of biochemical estimation of amino acids using ninhydrin

To evaluate free amino acids were extracted first from the dry samples with methanol. The free amino acids were quantified with ninhydrin method (Sun et al. 2006). Briefly, Amino acid solution (1 ml) and ninhydrin solution (1 ml) were put in screw-capped test tubes and heated in a boiling water bath for a pre-determined period of time. The schematic representation of the chemical reaction between amino acids and ninhydrin to develop purple color is represented in Fig. 1. After heating, the tubes were immediately cooled in an ice bath. Then, 5 ml of 50 % alcohol was added into each tube and thoroughly mixed with a Vortex mixer for 15 s. The absorbance (570 nm) of the reaction mixture was measured with a spectrophotometer (UV-2300, Japan). Two independent experiments each with duplicate samples were conducted and the reported values were the means of the two experiments. Linear regression was performed with commercial statistical software on a personal computer.

### Statistical analysis

All experiments were carried out in triplicate, and average values with standard deviation are reported. Mean separation and significance were analyzed using the Sigmaplot software package (Sigmaplot-11, USA). Correlation and regression analysis was carried out using the EXCEL program.

## Results and discussion

We have earlier reported the production of a keratinase from *P. woosongensis* TKB2 when grown in media containing only chicken feather as a sole nitrogen source (Paul et al. 2013). This indicated that the enzyme is a protease in nature and can deproteinize shrimp shell wastes.

### Zymogram analysis of crude enzyme

**Fig. 2:**
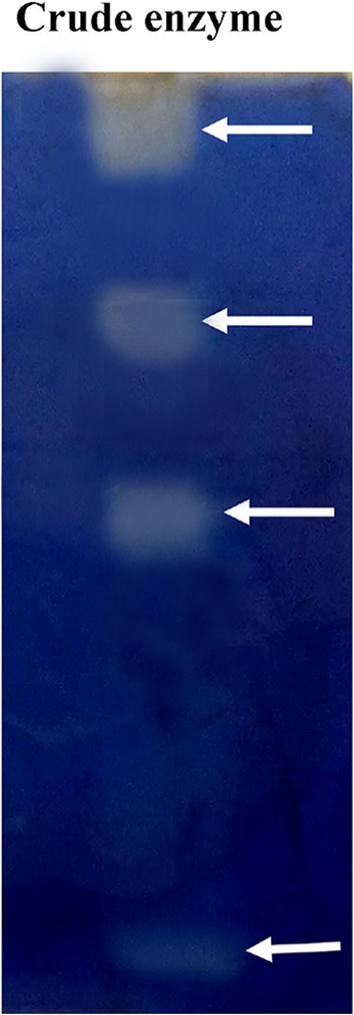
Zymogram of crude protease showed four distinguishable hydrolytic bands. *Arrows* indicate the protease hydrolytic bands

Crude enzyme showed four bands all identically protease activity (Fig. 2). This complied that the bacterial strain produced four protease enzymes that hydrolyzed the casein to produce clear zones on gel slab. These protease cocktails were used for deproteinization of Black tiger shrimp shell waste.

### De-carotenoid the BTSHW

The solvent extracted carotenoid was in the form of a paste with an orange–red color. The highest carotenoid yield (48.9 µg g^−1^ waste) from waste was obtained after 24 h of contentious agitation with hexane.

### Protein removal from BTSHW

Once the enzyme production was obtained, deproteinization of BTSHW was studied by incubation of these substrates with the crude cocktail protease of *P. woosongensis* TKB2. The percent of protein removal for BTSHW after 4-day incubation was 80 and 73.33 % for cocktail protease and commercial protease, respectively, whereas that of alkali- and acid-treated BTSHW was 91.66 and 61.89 %, respectively. By contrast, before optimization, 74.09 % deproteinization was observed with crude protease from BTSHW. The possible reason for the low protein removal for BTSHW may be a Maillard reaction associated with the cooking and high-temperature drying process. The Maillard reaction is known to render proteins (due to cross-linking with sugars and other compounds) resistant to protease treatment (Sikorski et al. 2009). The fact that acid-treated BTSHW did not yield a favorable result is possibly due to the destruction of many of the proteins during the treatment (Wang and Chio 1998).

In the present study, chitins obtained by deproteinization of shrimp shell wastes with crude enzyme or by chemical extraction were carried out and chemical compositions of chitins were compared (Table 1). High enzymatic deproteinization of BTSHW was achieved which reached 80 ± 0.4 % with an E/S ratio of 1:8 (wv^−1^) after 72 h incubation. Chemical deproteinization by NaOH (2 N) and HCl (2 N) of BTSHW was investigated at different incubation times (data not shown).

### Demineralization of BTSHW

In the recovery of chitin from shrimp shell waste, associated minerals should be removed as a second stage. The demineralization was completely achieved within 6 h at room temperature after treatment with 1.25 N HCl solutions at a ratio of 1:10 (wv^−1^). One of the factors determining the good quality of chitin is the low mineral content (Tolaimate et al. 2003). The four types of chitins obtained in this work contain metals significantly lower than those reported in other works (Percot et al. 2003; Chandumpai et al. 2004). The residual contents of Ca, Na, Mg, and K in four types of chitins were about 0.03, 0.012, 0.013, and 0.02 %, respectively.

The treatments employed to extract chitin from the shrimp shell wastes shown in Table 1 produced a mass of water insoluble white fibrous material, which indicated that a good yield for the chitin extraction was attained and no pigments were present in the chitin (Canizares et al. 2002). In fact, the discoloration of the chitin prepared by inversion of the two steps (i.e., deproteinization followed by demineralization) was less important. In addition, the HCl treatment may probably remove the residual acid-soluble proteins.

Attempts to compare the deproteinization effect found in this study with other reports were made. We found that the variation in analytical methods, calculation approach and demineralization made comparatively impractical. Nevertheless, in view of the shortage associated with the chemical treatment using a strong acid and base, the use of proteolytic enzymes to hydrolyze proteins associated with Black tiger shrimp shell waste to recover chitin was very promising.

### Optimization of process parameters

A response surface design is applied when the optimal region for running the process has been identified (Jain and Vigneshwaran 2012). RSM using Box–Behnken design was applied to determine the optimal levels of the three selected variables which significantly influenced deproteinization process. The respective coded (−1, 0, +1) and un-coded values of the variables at various levels are as follows—pH −8.5, 9.0, 9.5; temperature (°C)—40, 50, 60 and agitation speed (rpm) of 80, 100, 120. To study the combined effect of these variables, experiments were performed using different combinations. Table S1 summarizes the Box–Behnken experimental design along with the experimental and predicted responses from each individual experiment. By applying multiple regression analysis on the experimental data, the following second-order polynomial equation was found to describe deproteinization experiment:

7 $$\begin{aligned} &\text{Y}_{{\text{Deproteinization} \,(\% )}} = + 74.08 + 4.76 \times \text{A} + 0.63 \times \text{B} \\ & \quad + 11.07 \times \text{C} - 4.40 \times \text{A} \times \text{B} - 7.76 \times \text{A} \times \text{C} + 0.54 \times \text{B} \times \text{C} - 24.13 \, \\ & \quad \times \text{A}^{2} - 27.48 \times \text{B}^{2} - 4.95 \times \text{C}^{2} \\ \end{aligned}$$

The statistical significance of the Eq. 7 was checked by the *F* test. The analysis of variance (ANOVA) for response surface quadratic model is summarized in Table S2. The model *F* value of 113.27 for deproteinization process implies that the model was significant, and the coefficient of variation (*R* ^2^ value) ensured a satisfactory adjustment of the quadratic model to the experimental data and indicated that approximately 95 % of the variability in the dependent variables (responses) could be explained by this model.

The *P* value denoting the significance of the coefficients was also important in understanding the pattern of the mutual interactions between the variables. In this case, independent variables (A, B, C) and the quadratic terms of the factors (A^2^, B^2^, D^2^) were significant model terms.

The predicted sum of squares (PRESS) which is a measure how a particular model fits each point in the design was 844.24 for response. The adequate precision value which measures the ‘signal-to-noise ratio’ was found to be >4 which indicates an adequate signal. Thus, it can be concluded that the model was statistically sound and can be used to navigate the design space.

**Fig. 3:**
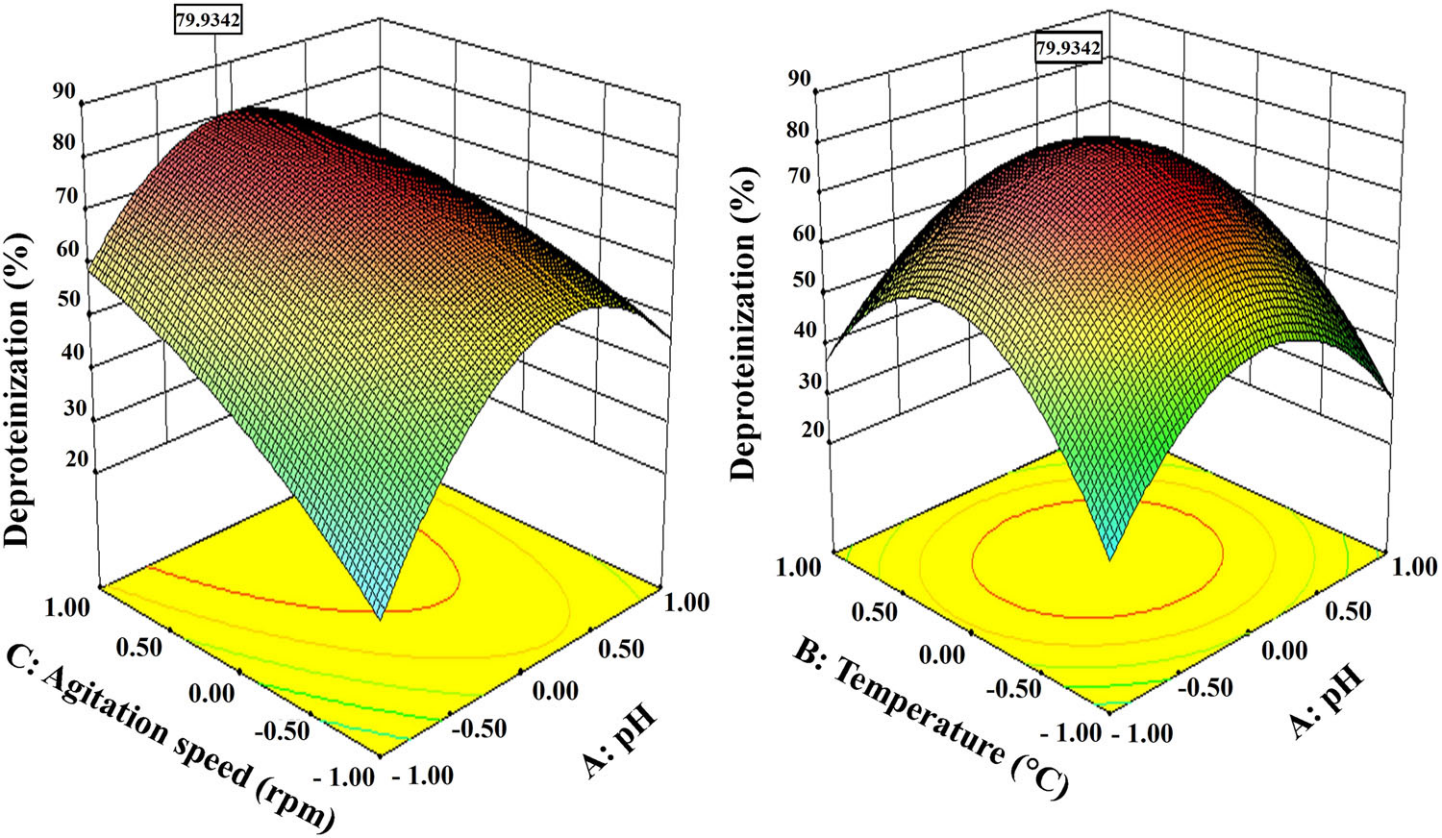
Box–Behnken response surface design curves for deproteinization with combination of agitation speed vs pH and temperature vs pH

To determine the optimal levels of the variables, 3D contour plots (Fig. 3) for deproteinization process were constructed according to the Eq. 4. To depict the interactive effects of independent variables on responses, two variables were kept constant while the other two variables varied within certain ranges. These contour plots provided a visual interpretation of the interaction between two factors, namely, pH and agitation speed followed by pH and temperature to facilitate the location of optimum experimental conditions (Jain and Vigneshwaran 2012). In this experiment the plots had elliptical contours enclosing the region of maximum activity within the experimental range.

The optimized variables were found using a desirability objective function that assigns relative importance to the responses. Solutions with higher desirability gave an optimum pH (8.82), temperature (50.05 °C) and agitation speed (100.98 rpm). Under these conditions, confirmation experiments were conducted in three replicates. The maximum protein yield of 79.93 % was observed. The statistical model produced 1.1-fold increased deproteinization than that in un-optimized state, which also showed the acceptability of the model for better deproteinization by enzymatic protease cocktail yield. The result indicates better deproteinization than some current reports (Oh et al. 2000; Wahyuntari and Junianto Setyahadi 2005), though very few higher yields had been also reported (Younesa et al. 2012). Our reports envisage a shorter period of treatments in which further treatments such as solicitation (de Vasconcelos et al. 2011) are not required.

### Chitin characterization

The characteristics of the raw BTSHW and chitins that were prepared by enzymatic and chemical (acid and alkali) treatments are shown in Table 1. The ground BTSHW before treatment contained a relatively high content of protein (48.16 ± 0.20 %), chitin (34.23 ± 0.25 %) and ash (30.66 ± 0.35 %). These results are comparable with those reported by previous studies (Manni et al. 2010; Younesa et al. 2012). The demineralization experiments were used in this study to reduce the mineral content to permissible limits in the chitin. Indeed, the ash content was reduced to about 0.83 %. This was lower than that found by Sini et al. (2007) and Younesa et al. (2012). Further, low-ash content for both chitins indicated the suitability of removal of calcium carbonate and other minerals from the raw BTSHW. There were no significant differences in the moisture content and ash among the four chitins prepared (*p* > 0.05). In contrast, the protein content was significantly higher in the chitin isolated with enzymatic deproteinization both by commercial and cocktail protease (*p* < 0.05) than chemical treatments. It is time that the enzymatic process we have adopted does not completely remove the residual protein associated with BTSHW. However, the residual protein on the instant case is much lower than usually found in the literature.

**Table 1:** Properties of the chitins obtained by deproteinization with crude proteases, commercial protease, alkali and by acidic methods

Percentage (%)	Raw BTSHW	Chitin 1 (cocktail protease)	Chitin 2 (commercial protease)	Chitin3 (acid treatment)	Chitin 4 (alkali treatment)
Moisture	65.06 ± 0.35	4.05 ± 0.05	4.58 ± 0.34	5.70 ± 0.35	3.51 ± 0.37
Ash	30.66 ± 0.35	0.83 ± 0.06	1.18 ± 0.34	3.68 ± 0.53	1.06 ± 0.02
Chitin	34.23 ± 0.25	26.43 ± 0.44	21.21 ± 0.21	10.19 ± 0.17	27.28 ± 0.27
Protein	48.16 ± 0.20	20.07 ± 0.07	26.69 ± 0.17	38.11 ± 0.12	8.29 ± 0.27
Lipid	5.2 ± 0.20	–	–	–	–
Appearance	–	White flakes	Slight yellowish flakes	Yellowish flakes	Yellowish flakes
Chitin recovery (%)	–	77.23 ± 0.21	62.06 ± 0.12	29.93 ± 0.05	79.83 ± 0.17

Although the deproteinization percentage is lower than that of chemical treatment, enzymatic deproteinization helps to avert many drawbacks of chemical treatment such as heavy metal residues, over-hydrolysis, break down of chitin, etc. Needless to mention, heavy metals referred are potential bio-hazards.

### Analysis of superficial characteristics by scanning electron microscopy (SEM)

Scanning electron microscopy images of BTSHW after DP by enzymatic and chemical treatments are shown in Fig. S1. The smashed BTSHW displayed smooth microfibrillar crystalline structure and left layer structure largely intact (Fig. S1a), while the SEM images of BTSHW after DP showed a slight fracture (Fig. S1b–e). The HCl treated BTSHW showed a hazy layer on the surface of BTSHW, which may be due to denatured proteins and minerals (Fig. S1b). The DP under the optimal conditions using alkali treatment and commercial enzyme treatment left layers stacked and showed signs of perforation with some depositions of mineral compounds (Fig. S1c, d). The BTSHW processed by cocktail enzyme and commercial chitin was morphologically similar (Fig. S1e and f). However, the Black tiger shrimp shell fragments became highly perforated and spongy after crude enzymatic treatment. It was apparent that the crude enzymatic treatments improved DP and DM efficacy and produced chitin polymer (Liu et al. 2012).

### FT-IR analysis of BTSHW

**Fig. 4:**
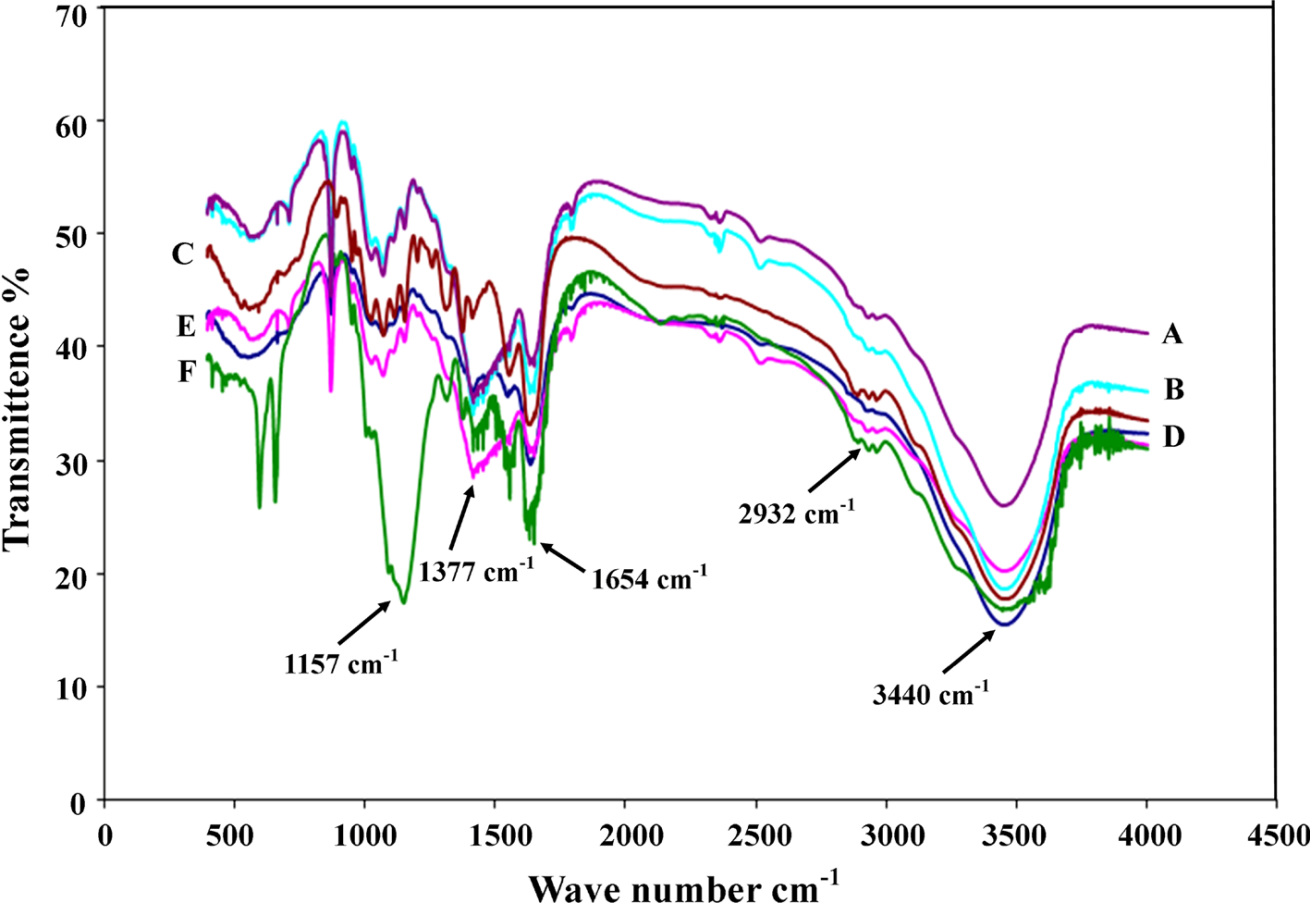
FT-IR spectra of commercial chitin (**a**), chitin from commercial protease (**b**), chitin from crude protease (**c**), from NaOH treatment (**d**), raw BTSHW and treatment with HCl (**e**) in this study

The differences in the FT-IR scans among all samples are displayed in Fig. 4. The spectra were characterized by three bands at 1,377, 1,654, and 2,932 cm^−1^, which corresponded to the vibrations of –NH, –C–O, and –CO–CH_3_ group, respectively (Liu et al. 2012). The bands between 890 and 1,156 cm^−1^ represented polysaccharide structures. It was notable that the bands at 1,654 and 1,377 cm^−1^ for other samples were more intense than that of commercial chitin (Fig. 4a), which confirmed the existence of chitin (Moreira et al. 2011). Compared to the commercial chitin, the band observed at 2,932 cm^−1^ demonstrated an intensification of the peak (Fig. 4b–e), and suggested the occurrence of deacetylation. Extent of deacetylation of the samples after cocktail enzymatic treatment, commercial protease, acid and alkali (chemical treatment) was 82.25, 80.34, 55.25 and 84.46 %, respectively (Table 2).

**Table 2:** DA and crystallinity index of commercial chitin, chitin obtained from crude protease, commercial protease, alkali and acid treatments

Samples	DA (%)	Crystallinity index (%)
Commercial chitin	88.10 ± 0.11	97.9
Chitin obtain from cocktail protease treatment	82.25 ± 0.07	88.0
Chitin obtain from commercial protease	80.34 ± 0.11	–
Chitin obtain from acid treatment	55.25 ± 0.06	–
Chitin obtain from alkaline treatment	84.46 ± 0.14	–

### X-ray diffraction analysis

**Fig. 5:**
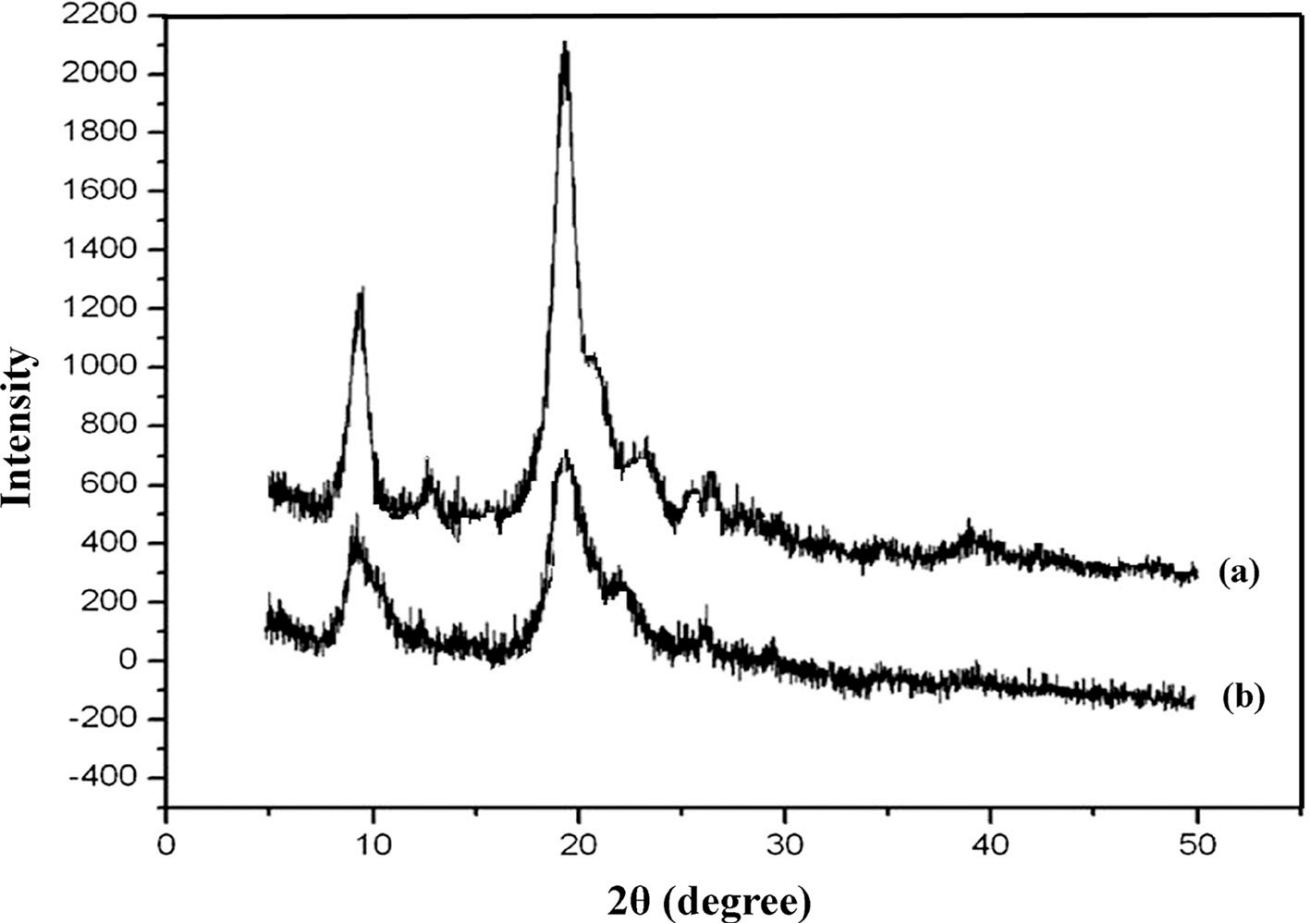
X-ray diffractogram of commercial chitin (**a**) and chitin from crude protease treatment (**b**) in this study

The crystallinity indexes of commercial chitin and chitin extracted by crude protease treatment were determined from the scattering intensity at two angles, one at 2θ = 9–10° and another at 2θ = 19–20° (Fig. 5). The results were in agreement with the literature, in which the purified chitin had a wide-angle X-ray diffraction pattern and showed two crystalline peaks at 2θ = 9.3 and 19.1° (Yen and Mau 2004). Similarly, Yen and Mau reported that fungal chitin displayed two crystalline reflections at 5.4–5.6 and 19.3–19.6° (Yen and Mau 2007). The crystallinity indices of commercial chitin and chitin obtained from cocktail protease treatment were 97.9 and 81.0 %, respectively (the baseline at 2θ = 16°). Overall, it was found that the application of protease cocktail reduced the crystallinity of chitin from 97.9 % in commercial chitin to 88.0 % in enzymatic treatment (Table 2). A lower crystallinity of polysaccharides indicates disruption of intra- and inter-molecular hydrogen bonds, in turn provides the possibility for more efficient chemical modifications in subsequent processing steps (Yen et al. 2009). The X-ray diffractograms of chitin powder obtained from crude protease treatment showed narrowed peak areas than the commercial chitin, confirming that further purification is necessary to obtain satisfactory chitin extractive.

### Detection of chemical shifts in BTSHW and comparison with chitin

NMR is one of the most reliable instruments in the examination of polysaccharide composition and sequential structure. NMR is a non-destructive method ensuring in retained structure and conformation of the polysaccharide, making it possible to monitor reactions and other structural and physical properties under different solvent conditions. Solid state ^13^C CP/MAS-NMR is known to be very sensitive to changes in the local structure. ^13^C CP/MAS-NMR spectrum of the chitin prepared by enzymatic deproteinization and commercial chitin is shown in Fig. S2. NMR analysis of the treated shrimp shell waste chitin gave similar peak pattern with that of commercial chitin. There are eight signals for the eight carbon atoms of chitin. The C_1_–C_6_ carbons of *N*-acetylglucosamine monomeric unit are shown between 50 and 110 ppm, indicating the high structural similarity. The carbonyl group is around 173 ppm, while the methyl group of the acetyl group produced a peak at around 23 ppm. The lesser intensity of the peaks of carbonyl and acetyl groups suggests that the deacetylation reaction is complete. Fig. S2 shows the chitin spectrum, in which the deacetylation of chitin is evident, since there are tight peaks at 23 and 173 ppm that correspond to the CH_3_ and C=O groups, respectively. The other peaks correspond to C_1_ (δ104.37), C_2_ (δ60.71), C_3_ (δ73.69), C_4_ (δ83.40), C_5_ (δ75.88), and C_6_ (δ55.33). As we have no other picks, it may be argued that the product was significantly pure and the removal of protein during extraction was almost total (Younesa et al. 2012). The presence of some background noise may be accounted for the presence of a possible by-product or some impurity in the sample, especially in commercial chitin (Fig. S2). The low-protein content is considered as a good indicator for purity of the final product.

### Oligopeptides and amino acid separation and estimation

**Fig. 6:**
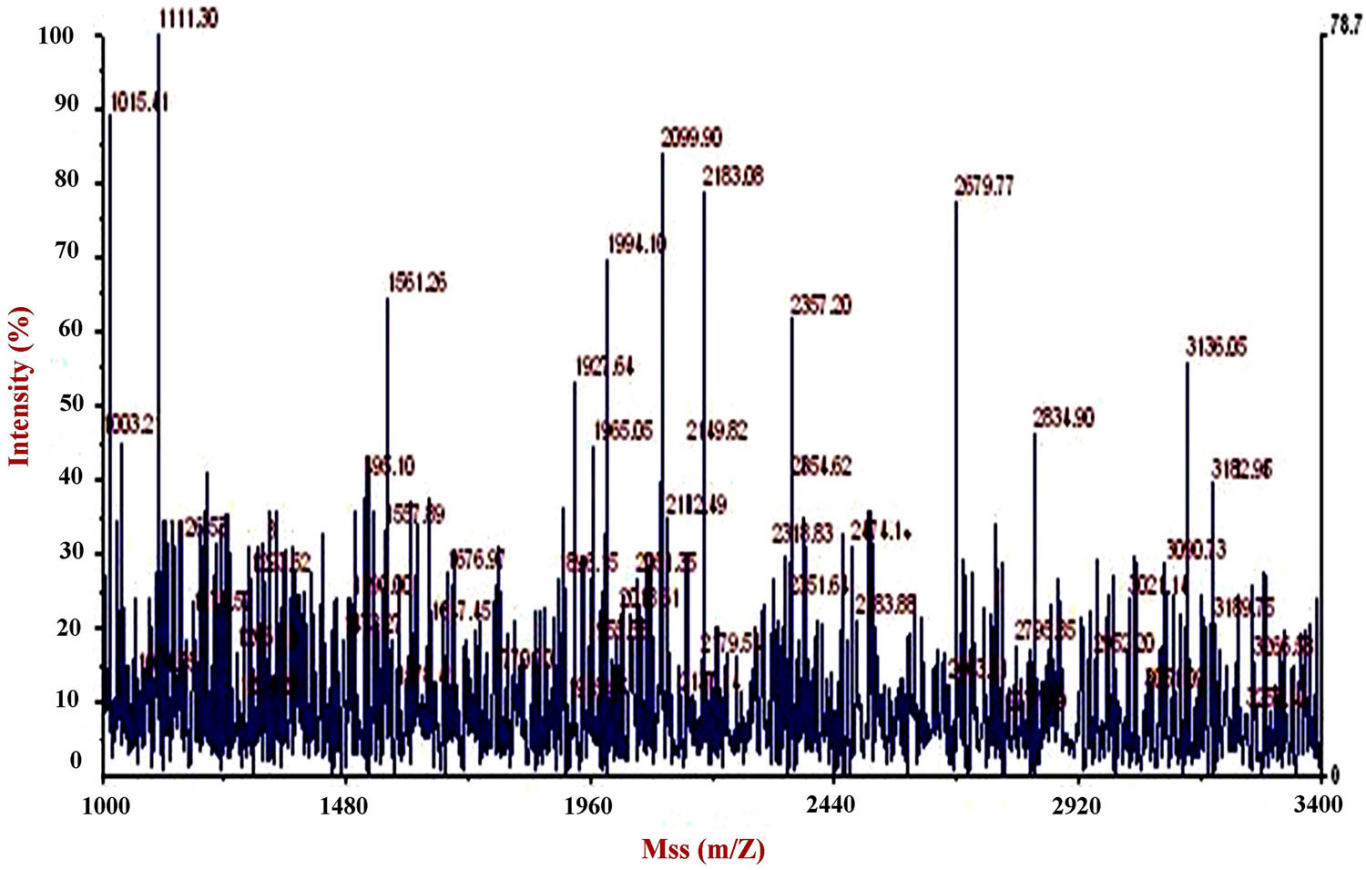
MALDI-TOF mass spectrum for oligopeptides present in treatment spent hydrolysate

In response to the increase in the amount of soluble protein in the culture, oligo or small peptide contents of the supernatants were analyzed to determine whether the increase in soluble proteins was derived from BTSHW. The culture supernatants were subjected to MALDI-TOF mass spectrometric analysis to survey the distribution of the molecular masses of the solubilized products. As shown in Fig. 6, numerous but specific signals were observed in the sample after fermentation for 4 days revealed multiple peaks from 1,015 to 3,182 *m/z*, indicating the presence of peptides generated by protein hydrolysis. For example, the thirteen sharply signal peaks (*m/z* = 1,015.41, 111.30, 1,561.26, 1,927.64, 1,965.05, 1,995.40, 2,099.90, 2,183.08, 2,357.30, 2,679.77, 2,834.90, 3,136.05, and 3,182.96) were observed. From this mass analysis, it is clear that molecular masses must be derived from the degradative products of shrimp shell.

The colorimetric method proposed allows simultaneous analysis of free amino acids in fermented broth. The concentration of the amino acids standard solution injected was 16.9 µg ml^−1^.

## Conclusions

This study has established that fermentation using a protease cocktail of *P. woosengensis* TKB2 alone gives chitin with satisfactory DM and DP efficacy and the resultant chitin has similar physicochemical and structural properties to commercial chitin. The oligopeptides and amino acid-rich deproteinized liquor should be used for feed and medium supplement. The enzyme method is a relatively simple profitable and environment-friendly alternative to the chemical method. This study at shaking flask under laboratory conditions may be suitable for large-scale operations also. RSM model significantly influences the DP process and enhances the DP rate of 1.1-fold. Moreover, this study gives yields satisfactory result for DP for chitin production at shake flask level. The issue of large-scale production of chitin through DP may be taken up for future studies.

## Electronic supplementary material

Below is the link to the electronic supplementary material.

Supplementary material 1 (DOC 3112 kb)
