# Erratum to: Production of chitin and bioactive materials from Black tiger shrimp (*Penaeus monodon*) shell waste by the treatment of bacterial protease cocktail

**DOI:** 10.1007/s13205-014-0250-9

**Published:** 2014-09-26

**Authors:** Tanmay Paul, Suman K. Halder, Arpan Das, Kuntal Ghosh, Arpita Mandal, Pijush Payra, Prasenjit Barman, Pradeep K. Das Mohapatra, Bikas Ranjan Pati, Keshab C. Mondal

**Affiliations:** 1 Department of Microbiology, Vidyasagar University, Midnapore, 721102 West Bengal India; 2 Department of Aquaculture Management and Technology, Vidyasagar University, Midnapore, 721102 West Bengal India

## Erratum to: 3 Biotech DOI 10.1007/s13205-014-0245-6

In the original publication of the article, the spelling of the first author name “Tanmay Paul” was incorrectly published as “Tanamy Paul”. The correct author name is “Tanmay Paul”.

