# Supplementary material for: Production of chitin and bioactive materials from Black tiger shrimp (Penaeus monodon) shell waste by the treatment of bacterial protease cocktail
Source: 3 Biotech. 2014 Aug 28;5(4):483–93. doi: 10.1007/s13205-014-0245-6 (PMC4522719; doi:10.1007/s13205-014-0245-6)
Supplement: Supplementary file 1 — Supplementary material 1 (DOC 3112 kb) [file 13205_2014_245_MOESM1_ESM.doc]

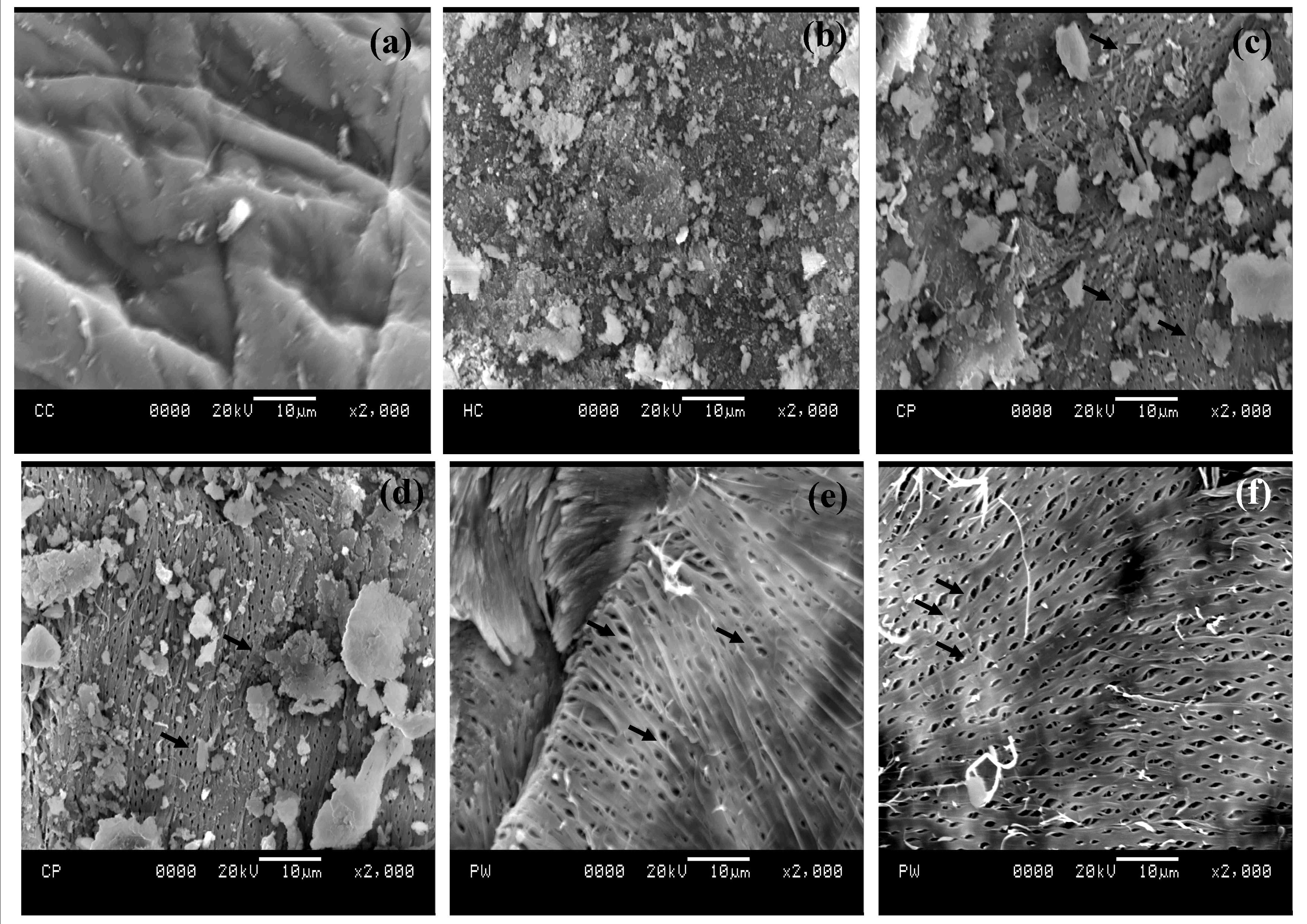
**Supplementary materials**

**Fig. S1.** SEM micrographs of Black tiger shrimp shell wastes (BTSHWs) (a), BTSHW after acidic treatment (b), BTSHW after alkaline treatment (c), BTSHW after commercial protease treatment (d), BTSHW after crude protease treatment (e) and commercial chitin (f) at 2000×magnification. (Arrows shows where the perforation occurred).

**

**

**Fig. S2.** ^13^CP/MAS NMR solid-state spectrogram of commercial chitin (a) and BTSHW deproteinized by cocktail protease (b).

**Table S1.** The actual design of experiments and response of deproteinization.

| **Run** | **Factor A: pH** | **Factor B: Temperature (°C)** | **Factor C: Agitation speed (rpm)** | **Response: experimental deproteinization (%)** | **Predicted deproteinization (%)** |
| --- | --- | --- | --- | --- | --- |
| 1 | 9 | 60 | 120 | 52.07 | 53.89 |
| 2 | 9.5 | 60 | 100 | 26.95 | 23.47 |
| 3 | 9 | 40 | 120 | 54.11 | 51.56 |
| 4 | 8.5 | 50 | 120 | 60 | 59.07 |
| 5 | 9 | 50 | 100 | 74.09 | 74.08 |
| 6 | 9.5 | 50 | 80 | 45.52 | 46.45 |
| 7 | 8.5 | 50 | 80 | 23.07 | 21.41 |
| 8 | 9 | 40 | 80 | 32.32 | 30.50 |
| 9 | 9 | 50 | 100 | 74.12 | 74.08 |
| 10 | 9.5 | 40 | 100 | 30.12 | 31.01 |
| 11 | 9 | 60 | 80 | 28.12 | 30.68 |
| 12 | 9 | 50 | 100 | 74.00 | 74.08 |
| 13 | 9 | 50 | 100 | 74.10 | 74.08 |
| 14 | 8.5 | 60 | 100 | 23.62 | 22.73 |
| 15 | 9 | 50 | 100 | 74.11 | 74.08 |
| 16 | 9.5 | 50 | 120 | 51.42 | 53.08 |
| 17 | 8.5 | 40 | 100 | 9.02 | 12.68 |

**Table S2.** Analysis of Variance (ANOVA) for the fit of experimental data to response surface model.

| **Source** | **Sum of squares** | **df** | **Mean square** | **F value** | **p-value Prob>F** | **Remarks** |
| --- | --- | --- | --- | --- | --- | --- |
| Model | 7685 | 9 | 853.91 | 113.27 | <0.001 | Significant |
| A-pH | 181.64 | 1 | 181.64 | 24.09 | 0.0017 |  |
| B-Temperature | 3.14 | 1 | 3.14 | 0.42 | 0.5394 |  |
| C-Agitation speed | 980.58 | 1 | 980.58 | 130.07 | <0.0001 |  |
| AB | 77.35 | 1 | 77.35 | 10.26 | 0.0150 |  |
| AC | 240.72 | 1 | 240.72 | 31.93 | 0.0008 |  |
| BC | 1.17 | 1 | 1.17 | 0.15 | 0.7058 |  |
| A2 | 2452.01 | 1 | 2452.01 | 325.24 | <0.0001 |  |
| B2 | 3179.46 | 1 | 3179.46 | 421.73 | <0.0001 |  |
| C2 | 103.15 | 1 | 103.15 | 13.68 | 0.0077 |  |
| Residual | 52.77 | 7 | 7.54 |  |  |  |
| Lack of Fit | 52.76 | 3 | 17.59 | 7548.51 | <0.0001 |  |
| Pure Error | 9.320E-003 | 4 | 2.330E-003 |  |  |  |
| Cor Total | 7738.00 | 16 | - |  |  |  |
